# Integrative Multi-Omics Analysis in Calcific Aortic Valve Disease Reveals a Link to the Formation of Amyloid-Like Deposits

**DOI:** 10.3390/cells9102164

**Published:** 2020-09-24

**Authors:** Marina A. Heuschkel, Nikolaos T. Skenteris, Joshua D. Hutcheson, Dewy D. van der Valk, Juliane Bremer, Philip Goody, Jesper Hjortnaes, Felix Jansen, Carlijn V.C. Bouten, Antoon van den Bogaerdt, Ljubica Matic, Nikolaus Marx, Claudia Goettsch

**Affiliations:** 1 Department of Internal Medicine I—Cardiology, Medical Faculty, RWTH Aachen University, 52074 Aachen, Germany; maugustoheus@ukaachen.de (M.A.H.); nmarx@ukaachen.de (N.M.); 2 Cardiovascular Medicine Unit, Department of Medicine, Karolinska Institute, 17177 Stockholm, Sweden; nikolaos-taxiarchis.skenteris@ki.se; 3 Department of Molecular Medicine and Surgery, Karolinska Institute, 17177 Stockholm, Sweden; ljubica.matic@ki.se; 4 Department of Biomedical Engineering, Florida International University, Miami, FL 33174, USA; jhutches@fiu.edu; 5 Department of Biomedical Engineering, Eindhoven University of Technology, 5600 Eindhoven, The Netherlands; d.c.v.d.valk@tue.nl (D.D.v.d.V.); C.V.C.Bouten@tue.nl (C.V.C.B.); 6 Department of Neuropathology, University Hospital, RWTH Aachen University, 52074 Aachen, Germany; jbremer@ukaachen.de; 7 Heart Center Bonn, Department of Medicine II, University Hospital Bonn, 53012 Bonn, Germany; Philip.Goody@ukbonn.de (P.G.); felix.jansen@ukbonn.de (F.J.); 8 Department of Cardiothoracic Surgery Division Heart and Lung, University Medical Center Utrecht, 3584 Utrecht, The Netherlands; j.hjortnaes@umcutrecht.nl; 9 Regenerative Medicine Center Utrecht, Utrecht University, 3584 Utrecht, The Netherlands; 10 Heart Valve Department, ETB-BISLIFE, 1940 Beverwijk, The Netherlands; a.vdbogaerdt@etb-bislife.org

**Keywords:** calcific aortic valve disease, multi-omics integration, transcriptomics, proteomics, amyloid structures

## Abstract

Calcific aortic valve disease (CAVD) is the most prevalent valvular heart disease in the developed world, yet no pharmacological therapy exists. Here, we hypothesize that the integration of multiple omic data represents an approach towards unveiling novel molecular networks in CAVD. Databases were searched for CAVD omic studies. Differentially expressed molecules from calcified and control samples were retrieved, identifying 32 micro RNAs (miRNA), 596 mRNAs and 80 proteins. Over-representation pathway analysis revealed platelet degranulation and complement/coagulation cascade as dysregulated pathways. Multi-omics integration of overlapping proteome/transcriptome molecules, with the miRNAs, identified a CAVD protein–protein interaction network containing seven seed genes (apolipoprotein A1 (APOA1), hemoglobin subunit β (HBB), transferrin (TF), α-2-macroglobulin (A2M), transforming growth factor β-induced protein (TGFBI), serpin family A member 1 (SERPINA1), lipopolysaccharide binding protein (LBP), inter-α-trypsin inhibitor heavy chain 3 (ITIH3) and immunoglobulin κ constant (IGKC)), four input miRNAs (miR-335-5p, miR-3663-3p, miR-21-5p, miR-93-5p) and two connector genes (amyloid beta precursor protein (APP) and transthyretin (TTR)). In a metabolite–gene–disease network, Alzheimer’s disease exhibited the highest degree of betweenness. To further strengthen the associations based on the multi-omics approach, we validated the presence of APP and TTR in calcified valves from CAVD patients by immunohistochemistry. Our study suggests a novel molecular CAVD network potentially linked to the formation of amyloid-like structures. Further investigations on the associated mechanisms and therapeutic potential of targeting amyloid-like deposits in CAVD may offer significant health benefits.

## 1. Introduction

Calcific aortic valve disease (CAVD) is the most prevalent valvular heart disease in the elderly population of the developed world, with a disease burden estimated to increase from 2.5 million in 2000 to 4.5 million in 2030 [1]. Calcification is deemed the hallmark of disease progression and a strong independent prognostic marker for adverse events in patients with asymptomatic aortic valve stenosis [2]. CAVD progression can be classified into two distinct phases [2]. The initiation phase is dominated by mechanical stress leading to endothelial cell injury, lipid deposition and a subsequent inflammatory response in the valvular endothelium [2]. This stage shares common pathophysiological mechanisms with atherosclerosis where inflammation coexists with microcalcification, facilitating the formation of hydroxyapatite crystals that together with lipid infiltration can accelerate disease progression [3,4]. The remodeling phase is characterized by fibrosis and an increasing rate of mineralization, leading to impaired valve function and elevated cardiac load that can lead to heart failure [3]. Current interventions for managing severe CAVD involve surgical aortic valve replacement or minimally invasive surgical transcatheter aortic valve implantation [5]. No therapeutic medication exists to halt or even slow CAVD progression [6]. 

Recent studies employed unbiased screening strategies on different molecular levels and generated large-scale datasets, to accelerate the discovery of novel underlying molecular disease mechanisms. The discovery of such mechanisms and biomarkers in single-omic studies benefits many areas of biomedical research, including cardiovascular biology [7]. Traditionally, analysis of disease networks is limited to surface interpretations, whereas a complete understanding of complex human disorders necessitates a full collection of its networks and the deconstruction of individual endophenotypes [8]. Such complex comprehension requires the integration of relevant big datasets and represents the great bottleneck in the cardiovascular field, limited to but a few studies [7]. Thus, we hypothesize that a transformative integration of multiple omic datasets into a biological-relevant context in CAVD may help to elucidate interactions across disease layers, supporting the construction of deeper molecular networks.

In this study, we analyzed transcriptome and proteome datasets from human CAVD to achieve differentially expressed molecules for downstream bioinformatics analysis. The application of bioinformatics tools accommodated the identification of altered pathways, enabling us to build a three-dimensional (3D) multi-omics layered structure of the CAVD and unveil novel insights into the CAVD pathobiology. To further strengthen and validate the association based on the multi-omics approach, we evaluated the findings using in vivo human CAVD and control tissue.

## 2. Materials and Methods

### 2.1. Study Selection and Data Collection

PubMed, Web of Science and EMBASE databases were searched (November 2019) using query ‘Medical Subject Headings’ terms for CAVD: “aortic valve stenosis OR heart valve diseases OR aortic valve calcification OR valvular calcification” in combination with a specific omic technique. The search terms for transcriptomics were: “microRNAs OR microRNA OR microRNA OR miR OR microRNA expression profile OR microRNA expression signature” and “gene expression profiling OR transcriptome OR RNA sequence analysis OR RNA expression profiling”. Proteomics were queried using “proteomics OR proteome” and metabolomics were obtained through searches for “metabolite OR metabolite profiling OR metabolome OR metabolomic pathways OR metabolomic network OR metabolomics”. There were no year restrictions. Overarching inclusion criteria were original studies on human data written in English that approached CAVD and proteomics, transcriptomics or metabolomics. We excluded studies on CAVD patients with bicuspid aortic valves because of a different pathophysiology and younger age compared to patients with tricuspid aortic valve [9]. Other exclusion criteria were rheumatic or mitral valve calcification, use of animals or cell samples and utilization of a targeted omics technique. The controls were non-diseased valves from autopsy, heart transplant or ascending aortic surgery and non-calcified areas adjacent to the calcified valve tissues as well as plasma from healthy subjects or patients with aortic regurgitation. All articles were screened by M.A.H. and N.T.S. In case of disagreement, C.G. was consulted.

Single-omics data were retrieved from four independent types of input: microRNA (miRNA), mRNA, protein and metabolite. Investigators were contacted in order to obtain missing data. The overall approach comprised four steps: (i) miRNAs/genes/proteins/metabolites were listed independent of the direction of regulation and considered as differentially expressed molecules in accordance to the cut-off value provided by the report (Supplementary Tables S1–S3). (ii) Proteins and mRNAs were converted to gene symbols using the UniProt database (https://www.uniprot.org). To bypass the heterogeneity between technical and statistical variances, when combining data from different approaches and cohorts, we selected only molecules that have been found differentially regulated in more than one study using data intersection by Venn diagrams (http://bioinformatics.psb.ugent.be/webtools/Venn) (Supplementary Table S4) [10]. (iii) Each single omics dataset was analyzed independently using pathway overrepresentation analysis. (iv) Datasets were combined through network-based multi-omics data integration. 

### 2.2. Pathway Over-Representation Analyses, Network Integration and Subcellular Localization 

The ConsensusPathDB database (http://consensuspathdb.org) was used for pathway analysis, employing the canonical pathways from the Kyoto Encyclopedia of Genes and Genomes (KEGG), Reactome and Biocarta [11]. To identify potential KEGG molecular pathway targets of miRNAs, an in-silico analysis was conducted using the DNA Intelligent Analysis (DIANA) Tools mirPath v.3 database (http://www.microrna.gr/miRPathv3) [12]. The use of predicted interactions derived from the DIANA-microT-CDS algorithm and the experimentally supported interactions derived from the DIANA-TarBase v7.0 target database, yielded experimentally supported as well as in-silico prediction of miRNA functional annotation. The Search Tool for the Retrieval of Interacting Genes/ Proteins (STRING) 11.0 database (https://string-db.org) was used to identify interacting proteins based on evidence of interaction. The web-based portal SubCell BarCode served for querying single gene subcellular localization (www.subcellbarcode.org) [13]. 

### 2.3. Construction of Layered Multi-Omics Network

Multi-omic data integration was performed using the web-based tool OmicsNet (https://www.omicsnet.ca) [14]. Genes and proteins were uploaded based on corresponding official gene symbol and miRNAs through corresponding miRBase IDs. IntAct (manually curated experimentally validated protein–protein interaction (PPI)) was considered for genes and miRNet (experimentally validated miRNA targets information based on TarBase and miRTarBase) for miRNAs. In case the network exceeded 3000 nodes, the minimum network setting (OmicsNet algorithm, which identifies the smallest subnetwork that connects all given nodes) was taken into account. Only interactors that targeted seeded nodes were considered. Function exploration was obtained by the network pathway analysis using KEGG and Reactome. MetaboAnalyst v4.0 (https://www.metaboanalyst.ca/) was used for integration of genes and metabolites with the network explorer function for metabolite–gene–disease interaction network based on the betweenness of the interacting molecules.

### 2.4. Human Tissue

In order to match the source of human non-diseased and calcified aortic valves to the omics studies, we included multicenter data and tissue. We selected the CAVD samples and controls in three ways: (1) Calcific and non-calcific portions of the same aortic valve leaflet provided by CAVD replacement surgery, (2) tissue bank-sourced aortic valves from CAVD replacement surgery and (3) control, non-diseased valves from heart donation.

Human calcified aortic valves were obtained from patients who underwent surgical valve replacement for severe aortic stenosis at the University Medical Center Utrecht, Netherlands, as part of routine surgery. Tissues were handed over anonymously, without any patient-specific information. According to the Dutch medical scientific research with human subjects act (WMO), secondary use of patient material does not require review by a Medical Ethics Examination Committee. Valve leaflets (*n* = 2 donors; 2 leaflets each) were divided into non-calcified (control) and calcified portions, embedded in optimal cutting temperature compound (OCT) and stored at −80 °C until use. Previous studies have shown that non-calcified regions of otherwise diseased tissues retain proteomic, transcriptomic and phenotypic characteristics of non-diseased aortic valve leaflets [15].

In addition, human calcified aortic valves were obtained from patients who underwent surgical valve replacement for severe aortic stenosis at the Heart Center Bonn, University of Bonn, Germany (*n* = 9 donors). Written informed consent was obtained from all patients. The local ethics committee approved the study (AZ 078/17). Valve leaflets were fixed in formaldehyde for 24 h, decalcified using Titriplex III-buffer (Merck, Darmstadt, Germany) for 72 h and paraffin-embedded. 

Furthermore, non-CAVD human aortic valve samples were also obtained from post-mortem donors, giving permission for research, according to national ethical and regulatory guidelines, maintained by the Dutch Transplant Foundation. The cause of death had not been related to valvular disease or conditions known to precede valvular disease. These valves were provided by ETB-BISLIFE, Heart Valve Department, Beverwijk, The Netherlands, which had assessed them to be unfit for implantation and were used in previously described studies [16,17]. Samples had been cryopreserved and stored in the vapor of liquid nitrogen within 48 h after circulatory arrest. Samples were transported in dry ice, temporarily stored at −80 °C, thawed, fixed overnight in formalin and embedded in paraffin. For this study, sections with a thickness of 10 μm were made from three valve samples that had shown no macroscopic signs of calcification. 

Control tissue (ligament flava, brain tissue with cerebral amyloid angiopathy) shown as a positive control for transthyretin and β-amyloid immunohistochemistry and Thioflavin S/Congo Red staining was obtained during routine diagnostic service. Showing these controls for research purposes is permitted by law in the state of North Rhine-Westphalia, Germany (§6 GDSG).

### 2.5. Immunohistochemistry

Frozen OCT-embedded tissues were cut into 10 μm sections and fixed in 4% paraformaldehyde (Sigma, Munich, Germany). For bright field immunohistochemistry, all cryo-sections were pretreated with 3% hydrogen peroxide solution (Roth, Karlsruhe, Germany) for 15 min to suppress endogenous peroxidase activity. After blocking in 4% of species-appropriate serum (Dako, Waldbronn, Germenay), sections were incubated with primary antibodies at 4 °C, overnight: human amyloid beta precursor protein (APP)/β-amyloid (1:100; Cell signaling technology; clone: NAB228, cat. 2450), human transthyretin (1:50; Invitrogen; clone: JM11-43, cat. MA5-32634), human alpha smooth muscle actin (1:100; Dako; clone: 1A4, cat. M0851) and human immunoglobulin G 1 (IgG1) (1:100; Dako; X0931). Biotin-labeled secondary antibodies (1:200, goat anti-mouse and anti-rabbit (Dako, Waldbronn, Germany)) were incubated for 45 min at room temperature. Sections were incubated for 30 min with ABC-Peroxidase solution (Vector Stain—ABC reagent, Vector Laboratories, Burlingame, CA, USA) followed by AEC (3-Amino-9-ethylcarbazole) Substrate Chromogen solution (Dako, Waldbronn, Germany). Slides were examined using a Leica DM 5500B microscope (Leica Biosystems, Nussloch, Germany).

Paraffin-embedded tissues were cut into 4 μm sections. Immunohistochemistry of paraffin sections was performed on a Dako Autostainer Link 48 (Dako, Waldbronn, Germany) using the EnVision Flex detection kit (Dako, Waldbronn, Germany) as described by the manufacturer with the following antibodies: β-amyloid (1:50; Dako, clone: 6F/3D; cat. M0872) and transthyretin (1:100; Dako; cat. A0002). For β-amyloid immunohistochemistry, paraffin sections were pretreated for 20 min at 95 °C in EnVision FLEX Target Retrieval Solution at low pH. Histological sections were viewed and imaged with an Axio Scope.A1 microscope (Zeiss, Oberkochen, Germany) using ZEN 3.1 software (Zeiss, Oberkochen, Germany). 

### 2.6. Thioflavin S and Congo Red Staining

Thioflavin S and Congo Red are two major histological stains used to detect any form of amyloid. The stainings were performed according to the protocol used for routine clinical diagnostics at the Department of Neuropathology at University Hospital Aachen, Germany. For Thioflavin S staining, hydrated paraffin sections were stained for 5 min in Mayer′s hemalum solution, washed with water for 5 min and then stained with 1% Thioflavin S solution (*w*/*v* in ddH_2_O; Sigma, Munich, Germany) for 5 min. Staining was differentiated in 70% ethanol, rinsed in ddH_2_O and mounted in glycerol-gelatin. Thioflavin S bound to amyloid emits green fluorescence. 

For Congo Red staining, hydrated paraffin sections were stained for 10 min in Mayer′s hemalum solution, washed with water for 10 min, incubated in fresh 1% NaOH solution (*w*/*v* in 80% ethanol, Merck, Darmstadt, Germany) for 20 min and stained with 0.5% Congo Red solution (*w*/*v* in 80% ethanol/0.1% NaOH, Merck, Darmstadt, Germany) for 20 min. Staining was differentiated in 100% ethanol, and mounted in vitroclud. Histological sections were viewed and imaged with an Axio Scope.A1 microscope (Zeiss, Oberkochen, Germany) using ZEN 3.1 software (Zeiss, Oberkochen, Germany).

### 2.7. Von Kossa Staining

Von Kossa silver stain was used to visualize inorganic phosphate crystals. Cryo-sections were incubated with 5% silver nitrate (Morphisto, Frankfurt am Main, Germany) for 30 min under ultraviolet (UV) light, then washed with 5% sodium thiosulfate (Morphisto, Frankfurt am Main, Germany). Nuclei were stained with nuclear fast red (Morphisto, Frankfurt am Main, Germany). Slides were examined using the Leica DM 5500B (Leica Biosystems, Nussloch, Germany).

### 2.8. Statistical Analysis

For pathway over-representation analysis with the ConsensusPathDB tool, the Fisher’s exact test was applied for computing significance of the annotation sets with respect to input molecules. Pathways adjusted q-value < 0.05 were considered as significantly enriched. For DIANA Tools mirPath v.3, the *p*-value and microT thresholds were defined as <0.05 and <0.08, respectively. For STRING, the combined STRING-score between protein >0.4 was applied. Function exploration of the network analysis from OmicsNet software applied hypergeometric tests and the built-in knowledgebase gene sets from KEGG and Reactome. A false discovery rate (FDR) value < 0.05 was considered significant. In Table 1 and Supplementary Table S5, data are presented as mean ± standard deviation (SD) and statistical significance among the variable age was calculated using an unpaired Student’s *t*-test. A *p*-value < 0.05 was considered statistically significant.

## 3. Results

### 3.1. Identification of Studies 

**Figure 1:**
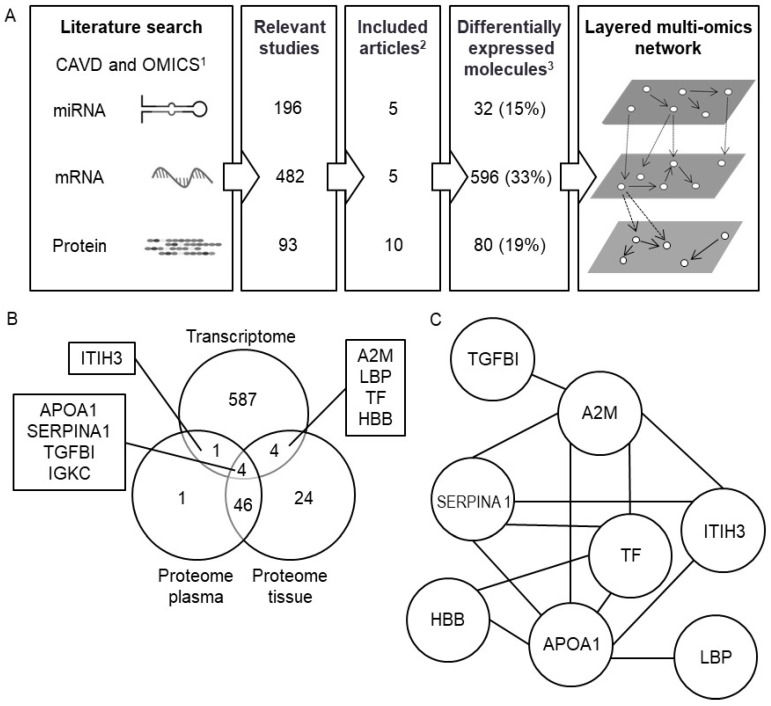
Dysregulated molecules in CAVD. (**A**) Workflow of the study. Literature search was performed for calcific aortic valve disease (CAVD) and omics. ^1^ microRNA (miRNA) and mRNA transcriptomics and proteomics. ^2^ Number of included articles after applying exclusion criteria. ^3^ Differentially expressed molecules in more than one study from miRNA, mRNA and protein single-omic studies. % indicate differentially expressed molecules from the total retrieved. (**B**) Venn diagram of the overlapping molecules between proteome and transcriptome datasets from plasma and tissue. (**C**) Protein–protein interaction (PPI) network of overlapping molecules between proteome and transcriptome using Search Tool for the Retrieval of Interacting Genes/Proteins (STRING). Immunoglobulin κ constant (IGKC) did not interact in the STRING network analysis. Apolipoprotein A1 (APOA1), β-globin (HBB), transferrin (TF), α-2-macroglobulin (A2M), transforming growth factor β-induced protein (TGFBI), serpin family A member 1 (SERPINA1), lipopolysaccharide binding protein (LBP), inter-α-trypsin inhibitor heavy chain 3 (ITIH3), immunoglobulin κ constant (IGKC).

The initial search yielded 196 unique records for miRNA transcriptomics, 482 for mRNA transcriptomics and 93 for proteomics in CAVD. After applying the inclusion and exclusion criteria, 5 miRNA transcriptomics [18,19,20,21,22], 5 mRNA transcriptomics [15,20,23,24,25] and 10 proteomics [15,26,27,28,29,30,31,32,33,34] records were eligible for the subsequent analyses (Figure 1A, Supplementary Figure S1, Supplementary Tables S1–S3). We identified only one single metabolomics study, which was only considered for integration analysis. The studies included 172 samples from CAVD patients and 197 from controls subjects. The baseline characteristics from the cohorts are presented in Table 1 and Supplementary Table S4. The CAVD group was 9.3 years older than the control group (*p* = 0.008). 

### 3.2. Pathway Over-Representation Analysis 

Analysis of molecules with altered expression profile between CAVD and control that were detected in at least two independent single-omic studies revealed 32 miRNAs, 596 mRNAs and 80 proteins (Supplementary Table S5)—representing 15%, 33% and 19% from the initial input of miRNA, mRNAs and proteins, respectively (Figure 1A). Three of the 32 miRNAs were present in three different articles, 44 of the 596 mRNAs were present in four articles, and five of the 82 proteins were common in more than five articles. Lumican was most frequent, appearing in seven articles (Supplementary Table S5, Figure S2).

We performed pathway over-representation analyses to identify enriched pathways in CAVD. First, the single-omic groups were investigated individually. A total of 61, 171 and 107 significantly overrepresented pathways were identified from the analyses of miRNA, mRNA and proteins, respectively. The top 3 over-represented pathways for miRNA were: proteoglycans in cancer, protein process in endoplasmic reticulum and viral carcinogenesis (Supplementary Table S6). The top 3 over-represented pathways for mRNAs included immune system, extracellular matrix organization and rheumatoid arthritis (Supplementary Table S7). The top 3 over-represented pathways for proteins featured complement/coagulation cascades, regulations of insulin-like growth factor transport and platelet degranulation (Supplementary Table S8). Since half of the proteomics studies were performed with tissue and the remaining with plasma, we analyzed the proteome from plasma and tissue separately and found similar results, with coagulation/complement cascade and platelet degranulation among the most significant over-represented pathways (Supplementary Tables S9 and S10).

### 3.3. Complement/Coagulation Cascade and Platelet Activation/Degranulation Pathways are Over-Represented after Multi-Omics Intersection

Whilst the global view provided by the overrepresented pathways led to a large number of pathways on single molecular level, it did not offer a focus on CAVD key regulators. Consequently, we tailored our analysis to common enriched pathways from mRNAs and proteins. Collectively, the mRNAs and the proteins from CAVD displayed 667 unique genes (Figure 1B). The pathway analysis revealed over-representation of the immune system, complement/coagulation cascades, extracellular matrix organization, integrin cell surface interaction and platelet degranulation pathways (Supplementary Table S11). To facilitate the interpretation, we reconstructed the pathway networks and highlighted the shared differentially expressed mRNAs and proteins within the complement/coagulation cascades and platelet activation pathways (Supplementary Figures S3 and S4). 

Analysis at the intersection of mRNAs and proteins revealed nine common genes: apolipoprotein A1 (APOA1), hemoglobin subunit β (HBB), transferrin (TF), α-2-macroglobulin (A2M), transforming growth factor β-induced protein (TGFBI), serpin family A member 1 (SERPINA1), lipopolysaccharide binding protein (LBP), inter-α-trypsin inhibitor heavy chain 3 (ITIH3) and immunoglobulin κ constant (IGKC) (Figure 1B), that, excepting IGKC, interacted in a PPI network (Figure 1C). Gene over-representation analysis supported our previous finding that platelet degranulation/activation/aggregation and complement/coagulation cascade are over-represented (Supplementary Table S12). 

Next, we analyzed the intersection between mRNAs and proteins from both plasma and tissue independently, and found four shared genes (APOA1, SERPINA1, TGFBI, IGKC) across the three datasets (Figure 1B). Plasma and tissue proteome shared most of the reported proteins and only clusterin (CLU) was exclusive for the plasma proteome.

### 3.4. Multi-Omics 3D Layered Network in CAVD 

To identify novel regulatory mechanisms in CAVD, we reconstructed the data in a multi-omics 3D layered network. Combining the original set of the 32 miRNAs, 596 mRNAs and 80 proteins unveiled an intrinsic complex network of 4147 nodes and 7394 edges that was trimmed to the minimum connected network (Supplementary Figure S5). The global view of multiple processes through over-represented pathways provided 119 (KEGG) and 120 (Reactome) pathways (Supplementary Tables S13 and S14). Of interest, platelet activation/degranulation emerged as significantly enriched.

**Figure 2:**
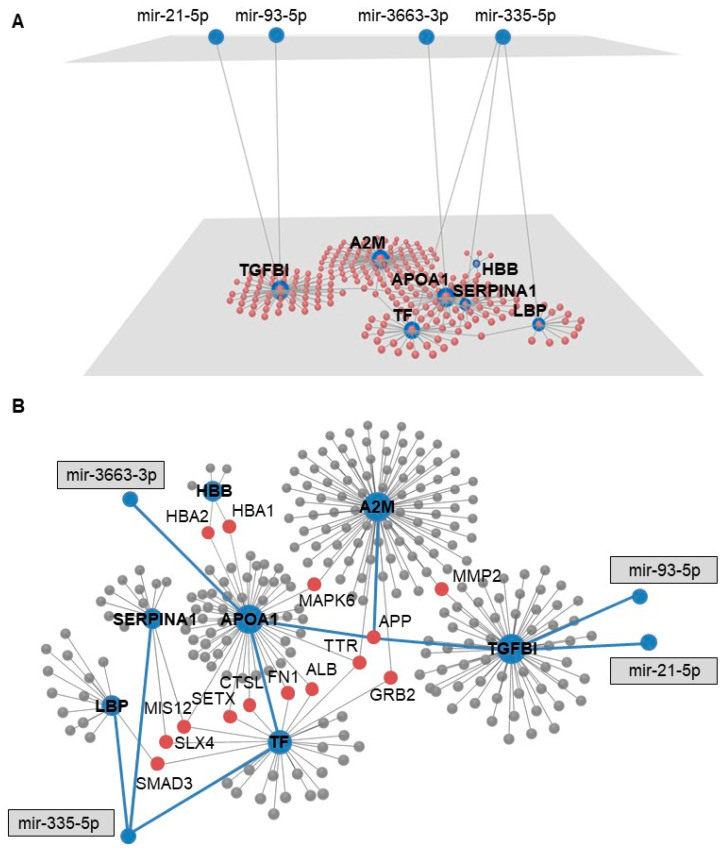
Three-dimensional (3D) multi-omics layered network in CAVD. (**A**) 3D multi-omics layered network containing differentially expressed miRNAs (upper layer) and overlapping molecules between transcriptome and proteome (lower layer). Blue nodes represent input molecules. Red nodes are based on protein–protein interactions (PPI). (**B**) Relevant PPI from A. Blue nodes: input molecules/miRNAs. Red nodes: connector molecules to at least two input molecules. Gray nodes: connector molecules. Blue line: network connecting the input molecules/miRNAs.

Next, the identified nine overlapping molecules between mRNAs and proteins were integrated with the 32 differentially expressed miRNAs, creating a highly connected novel 3D layered network. This network provided a PPI interactome of 7 input genes (APOA1, HBB, TF, A2M, TGFBI, SERPINA1, LBP) to 4 different input miRNAs (miR-335-5p, miR-3663-3p, miR-21-5p and miR-93-5p), with 256 nodes and 269 edges (Figure 2A). Moreover, the layered PPI network contained 14 connector genes directly connecting at least two input genes (Figure 2B, Supplementary Table S15). The amyloid beta precursor protein (APP) and transthyretin (TTR) connectors were connected to three input genes. Functional annotation of the 3D layered network revealed 49 and 14 over-represented pathways in KEGG and Reactome, respectively (Supplementary Tables S16 and S17). Consistent with the network from the original dataset, platelet activation/degranulation, response to elevated platelet cytosolic Ca^2+^ and TGF-beta signaling emerged as significantly overrepresented pathways. Exploration of the subcellular localization of the input and connector genes revealed cytoplasm, nucleus and extracellular space as primary localization sites (Supplementary Figure S6).

The integration of tissue-originated or plasma-originated proteins with the differential miRNAs maintained the connector molecules (including APP and TTR) and over-represented pathways (Supplementary Figure S7, Tables S18–S21). 

**Figure 3:**
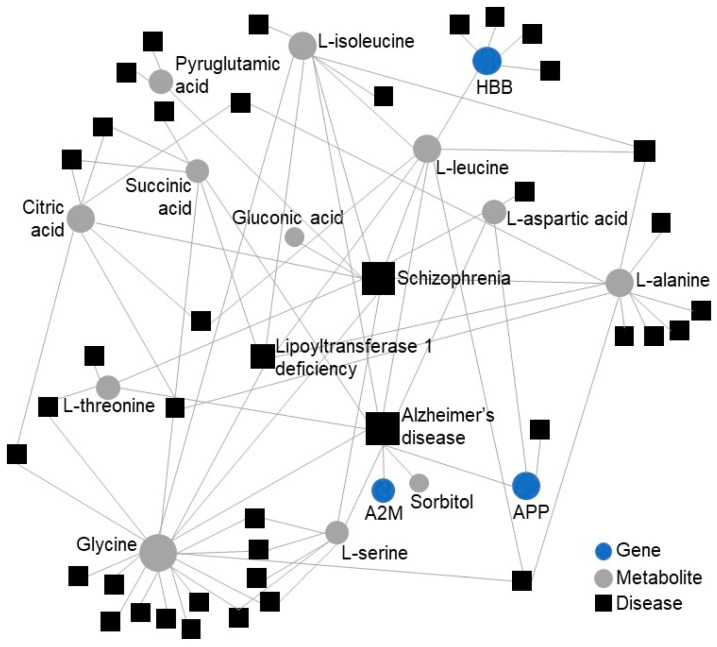
Metabolite–gene–disease interaction network in CAVD. Metabolite–gene–disease network based on the input of 19 differentially abundant metabolites and the 7 overlapping genes between transcriptome and proteome and its 14 connector genes from Figure 2B. The predicted network is based on the betweenness of the interacting molecules. The node size scales indicate the degree (connectivity) of nodes in the network. Gray nodes: metabolites. Blue nodes: input genes. Black squares: disease.

Finally, to provide a more thorough understanding of the disease process and the relevance of particular circulating factors, we added another molecular layer—the metabolome. We integrated the PPI interactome (7 input and 14 connector genes from Figure 2B) with the 19 differentially regulated metabolites reported in Mourino-Alvarez et al. [31]. The metabolite–gene–disease interaction network provided a PPI interactome of 3 input genes and 11 input metabolites (Figure 3). Alzheimer’s disease and schizophrenia exhibited the highest degree of betweenness in the network.

### 3.5. Calcific Aortic Valves Express Molecules Features of Amyloid Structures

**Figure 4:**
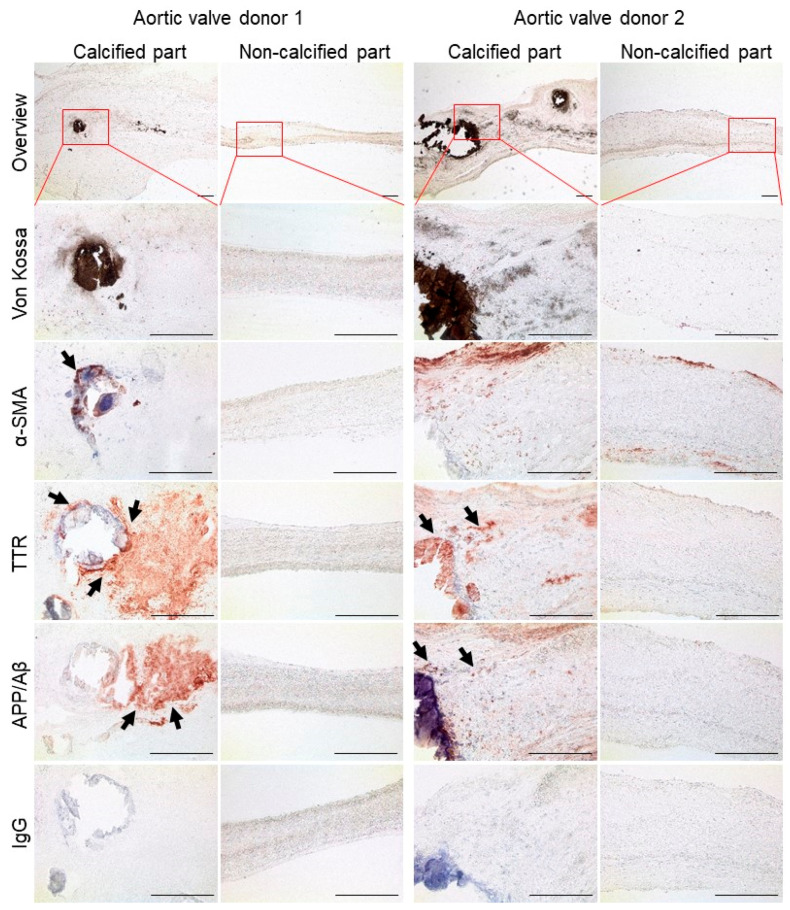
Human calcified aortic valves are transthyretin (TTR) and amyloid precursor protein (APP)/β-amyloid (Aβ)-immunoreactive. Aortic valves from patients with aortic stenosis were divided into the calcified and non-calcified portions. Calcification was visualized by von Kossa staining. Immunohistochemistry was performed for smooth muscle alpha-actin (αSMA), TTR, and APP/Aβ. Immunoglobulin G (IgG) served as control. One leaflet from each of two donors is shown. The first row shows an overview of the tissue and indicates the respective area of the magnified images. The arrows indicate positive signal. Bar: 200 μm.

Next, we assessed the presence of TTR and APP as well as amyloid structures in human CAVD tissue by immunohistochemistry and histology. Fibro-calcific regions of aortic valves were localized by a common cellular marker of leaflet remodeling, smooth muscle alpha-actin (αSMA), and von Kossa-positive calcification. Fibro-calcific regions of human aortic valves exhibit TTR and APP/β-amyloid (Aβ) immunoreactivity, while no expression was observed in non-calcified regions (Figure 4). Non-diseased aortic valves from donation also did not have detectable TTR and APP/Aβ expression (Supplementary Figure S8).

**Figure 5:**
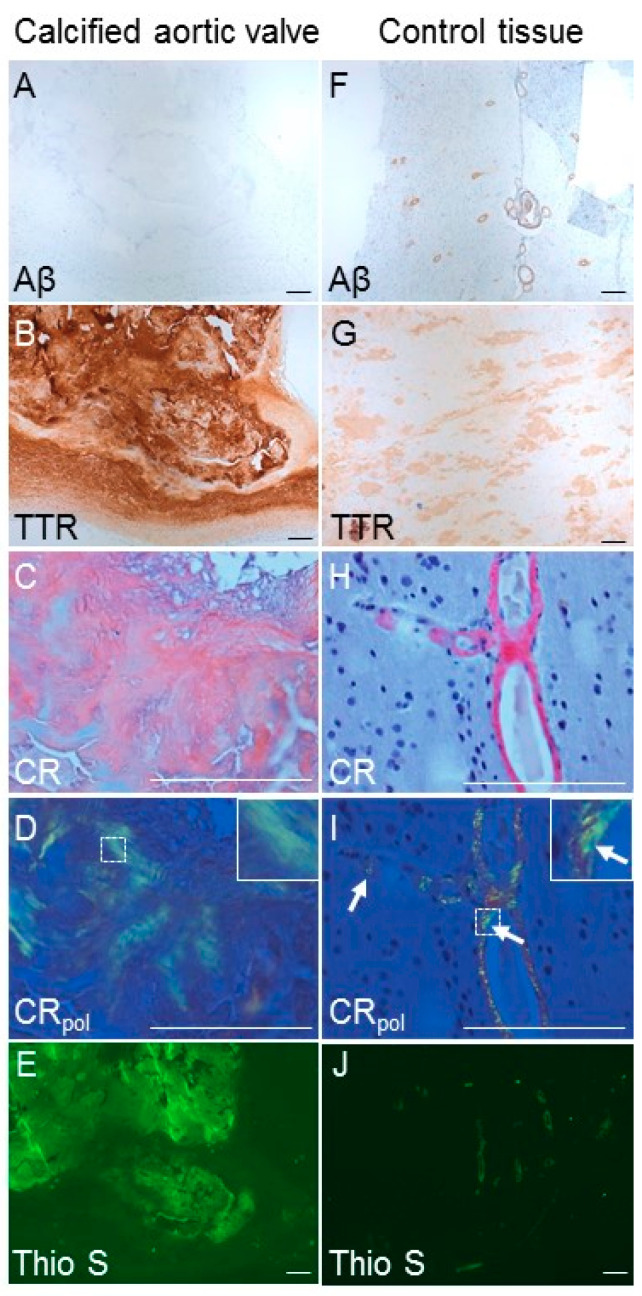
Transthyretin (TTR)-positive staining associates with Thioflavin S-positivity in human calcified aortic valves. Representative (**A**) β-amyloid (Aβ), (**B**) TTR, (**C**,**D**) Congo Red (CR—bright field, CR_pol_—polarized light) and (**E**) Thioflavin S (Thio S) fluorescence images of human calcified aortic valves (decalcified) of patient with aortic stenosis. One donor out of *n* = 9 is shown. (**G**) Positive control for TTR (amyloid within the ligamentum flavum). (**F**,**H**–**J**) Brain tissue with cerebral amyloid angiopathy as positive control for Aβ (**F**), Congo Red (**H**,**I**) and Thio S (**J**). Arrow in J indicates Congo Red-positive signal showing areas with the typical “apple-green birefringence” in polarized light. Insert in D and I shows a higher magnification of the dashed area. Bar: 200 μm.

Next, we assessed the presence of amyloid in calcified valves using Thioflavin S and congo red staining as well as by immunohistochemistry for TTR and Aβ—all used in clinical routine for detecting TTR-containing amyloid deposits and/or Aβ deposits. Although Aβ was undetectable in calcified aortic valves (Figure 5A), we observed TTR-immunoreactive regions (Figure 5B) that were partially congophilic and showed yellowish to slightly greenish birefringence after congo red stain in polarization microscopy (Figure 5C,D). Although amyloid is reported to typically display apple-green birefringence, amyloid frequently shows yellow birefringence [35] (Figure 5J). Nevertheless, since aortic valves are rich in collagen and elastin that are also birefringent and can be misinterpreted as amyloid [36], we stained the calcified aortic valves with a second amyloid-staining dye, thioflavin S. We found that TTR-immunoreactive regions were also partially thioflavin S-positive (Figure 5E), further supporting that TTR-immunoreactive deposits in calcified aortic valves display amyloid-like properties. TTR-containing-amyloid-rich ligamentum flavum and brain tissue with cerebral amyloid angiopathy were used as positive controls (Figure 5F–J).

## 4. Discussion

The present proof-of-concept study shows that bioinformatic-based re-analyses of multiple omic datasets can lead to novel disease mechanisms. We described a novel network in CAVD that associates to Alzheimer’s disease in the interaction analysis and demonstrated the presence of TTR-enriched amyloid-like deposits in calcified aortic valves. Moreover, our over-representation analyses indicated a pathway–phenotype correlation highlighted by the significant participation of coagulation/complement cascades, as well as platelet activation/degranulation pathways, aligning and strengthening the current state-of-the-art in the CAVD field. Single molecules within these pathways are shown to play a role in CAVD, for example thrombin, tissue factor, fibrinogen α chain, von Willebrand factor and complement C3 [37,38,39,40].

The coagulation/complement cascade pathway was enriched in four of the 20 input papers [15,28,30,31]; however, specific implications of the entire dysregulated pathway were not further discussed. Importantly, genes derived from tissue and plasma, suggesting that molecules within the coagulation/complement cascade pathway may originate from the circulation and be produced in situ by valvular resident cells, as occurs in atherosclerosis with myofibroblasts and/or macrophages [41].

The complement/coagulation-platelet crosstalk is highly recognized in innate immunity [42] and early atherogenesis [43]—our findings suggest that it could also drive CAVD. Patients with aortic stenosis display dysregulated platelet function [44]. Activated platelets accelerate the progression of aortic stenosis in mice by promoting the osteogenic transition of valvular interstitial cells [45]. Platelets are able to initiate the complement/coagulation cascade [46], while the coagulation pathway can activate platelets [47]. One interplaying factor—serotonin—was differentially expressed in the only CAVD metabolomics study [31]. Serotonin is released from platelets during blood clotting and promotes vasoconstriction. The serotoninergic system associates with platelet activation and the pathogenesis of aortic stenosis through valve fibrosis and ventricular remodeling [48]. An experimental study demonstrated that specific serotonin receptor (5-HT2B) activators potentiated aortic valve remodeling and dysfunction [49]. The association between serotonin signaling and general CAVD has not previously been elucidated. However, off-target activation of 5-HT2B has led to valve remodeling and the subsequent recall of several drugs, including cabergoline and fenfluramine [50].

Our multi-omic 3D layered network contained CAVD hallmark genes (SMAD3 [49], FN1 [51], CTSL [52], MMP2 [53]), supporting the proof-of-concept of our strategy. Applying the recently published molecular atlas of the aortic valve [15], we were able to map LBP and FN1 to the fibrosa, the calcification-prone layer of the aortic valve, and HBB, SERPINA1, APOA1, TF and ALB to the valve spongiosa layer. To date, the spongiosa layer has not been central to CAVD research. 

We identified a novel 3D network linking seven input genes (APOA1, HBB, TF, A2M, TGFBI, SERPINA1, LBP), four input miRNAs (miR-335-5p, miR-3663-3p, miR-21-5p and miR-93-5p) and two connectors (APP, TTR). The majority of these genes are known to participate in amyloid plaque formation and neurodegenerative disorders. Our novel network points towards a previously emerged link between cardiovascular disease and Alzheimer’s disease [54]. Indeed, Alzheimer’s disease revealed one of the highest betweenness in our multi-integration approach. This link was not observed in the single-omics studies. New emerging concepts lead to the hypothesis of a common pathogenesis of cardiovascular disease and Alzheimer’s disease either from a systemic or metastatic origin leading to multi-organ failure [54]. Recently, amyloid-β deposits, biochemically and structurally similar to those found in the typical Alzheimer’s disease pathology, were also recognized in the hearts of patients with idiopathic dilated cardiomyopathy [55]. This study demonstrated that Aβ pathology co-exists in the brain and the heart of patients with Alzheimer’s disease. Alzheimer’s disease patients with myocardial Aβ deposits showed compromised myocardial function and elderly patients exhibited increased aortic valve peak velocity, an indicator of aortic stenosis [55]. So far, there is limited epidemiological and clinical evidence for an association between CAVD and Alzheimer’s disease. Alzheimer’s disease patients have a higher frequency of aortic valve thickening and aortic regurgitation compared to age-matched controls [56]. ApoE4, a genetic risk factor for Alzheimer’s disease, is an independent predictor of CAVD [57]. However, the Rotterdam Study failed to show an association between the presence of CAVD with the risk of dementia or Alzheimer’s disease [58]. More research is needed to clinically evaluate the association between Alzheimer’s disease and CAVD. Whether CAVD and Alzheimer’s disease share common risk factors in a degenerative manner and/or whether their respective underlying mechanism are independent, functionally related and/or synergistic is a growing and intriguing research field given the increasing number of elderly patients with many comorbidities. This underscores the urgent need for a better understanding of how disease states in elderly multi-morbid patients influence the mechanism of aortic stenosis and CAVD initiation and progression, especially the underlying pathologies like fibrosis and calcification.

In our 3D network, APP and TTR were the two connector genes that interact with three input molecules, therefore presenting a central hub. Here, we demonstrated the presence of APP and TTR in calcified aortic valves—two proteins involved in the formation of amyloid structures. Of note, APP and TTR expression was absent in healthy aortic valves and the non-diseased portion of CAVD valves. APP is the precursor for Aβ, a major constituent of amyloid plaques found in the brain of patients with Alzheimer’s disease [59]. Aβ interacts with numerous molecules to fine-tune its function. Several of these interactors were present in our multi-omics network. APOA1 binding to APP affects the morphology of amyloid aggregates [60]. Binding of A2M and TF to the Aβ-peptide prevents amyloid plaque formation [61,62]. TTR, another amyloidogenic molecule, also present in tissue proteomics, has neuroprotective functions, and binding to Aβ suppresses its fibrillation [63]. Moreover, a complex of TTR, APOA-1 and C3 might be involved in Aβ clearance [64,65]. In addition, HBB and TGFBI were recently identified in Aβ-enriched extracts from Alzheimer’s patients [66]. Cumulative experimental evidence showed that Aβ interacts with different coagulation factors, promoting a prothrombotic and proinflammatory milieu [67,68,69]. Experimental studies showed that controlling the hypercoagulant state inhibits Alzheimer disease progression [70]. 

During platelet degranulation, platelets release APP from their alpha granules [71]. Platelet-derived APP stimulate pro-inflammatory signaling to promote atherosclerosis development [72] while APP-deficiency reduces atherosclerotic plaque size in mice [73]. Interestingly, selective overexpression of mutated human APP in the brain accelerated aortic atherosclerosis in ApoE-deficient mice [74]. APP is also present in the microvasculature surrounding advanced human carotid artery plaques [72]. The late stage of atherosclerosis might bear similarities to CAVD development, suggesting that in diseased aortic valves prone to calcify, APP can be released from activated platelets and may participate in the initiation of calcification. APP could act as a calcium nesting site in CAVD since its molecular structure has active binding sites for divalent ions like copper and zinc, resulting in Aβ–metal aggregates [75]. 

In calcified aortic valves, we detected TTR-positive deposits with amyloid-like properties based on congo red and thioflavin S staining. An association between amyloidosis and calcification was reported for aortic valves in autopsy studies. Calcified aortic valves showed congophilic, amyloid deposits associated to scar tissue [76,77,78,79]. Results of the congo red staining have to be taken with caution, since aortic valves are rich in collagen and elastin that are also birefringence and can be misinterpreted as amyloid [80]. 

Interestingly, patients with TTR cardiac amyloidosis showed a relatively high prevalence of moderate to severe aortic stenosis [81,82,83]. Clinical trials might determine whether amyloid-directed therapies recently developed for the treatment of TTR amyloid cardiomyopathy [84] are a therapeutic option for aortic stenosis.

**Figure 6:**
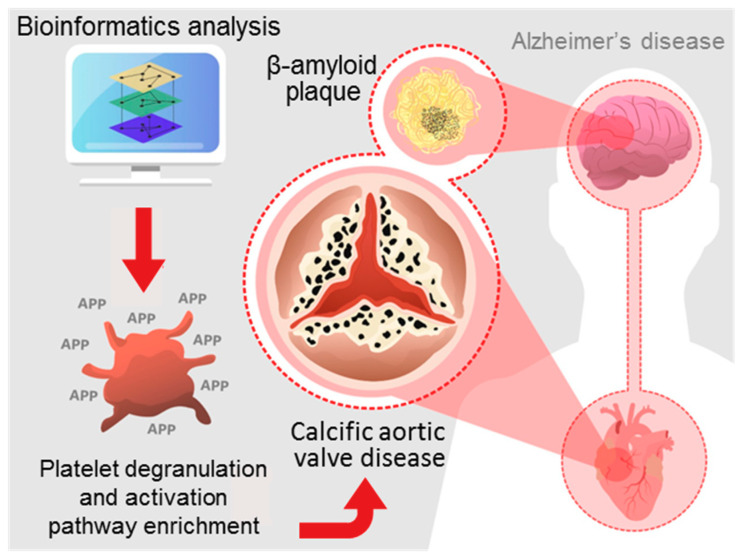
Our study provides a novel molecular CAVD network that is linked to the formation of amyloid-like deposits known from Alzheimer’s disease potentially though the platelet activation/degranulation pathway.

Overall, our data suggest molecular mechanisms of mineralization in CAVD that are potentially linked to the formation of amyloid-like deposits present in Alzheimer’s disease and cardiac amyloidosis that might involve platelet activation/degranulation (Figure 6).

Our study exhibits limitations. Firstly, the considerable heterogeneity between the publications used in this study. This explains why we identified only a minority of common molecules between the studies or between transcriptome and proteome. While all CAVD tissue samples came from patients who underwent aortic valve replacement, there was a variety in the source of non-calcified control aortic valve leaflets that might differently affect expression pattern. Moreover, the control group was significantly younger than the CAVD group. CAVD is—like Alzheimer’s disease—an age-related disease; therefore, our reported observations may reflect the age difference between the groups. Age-matched controls should be taken in consideration for further omics studies in the field [85]. Different normalization methods for omics data generate considerably different results [86]. In addition, the included studies had distinct sample quantities and cut-off thresholds that might add bias to certain studies. The heterogeneity drawback could explain why we did not observe a pattern between miRNAs and target genes through up- and down-regulation dynamics. Secondly, our current data integration method does not incorporate any attempt to statistically address the limitations and restricts the analyses to linear relationships among variables. Thirdly, our search strategy paired a high recall rate with a low precision rate [87], 562 records were excluded because they centered on other cardiovascular diseases, despite the “aortic valve” search term.

Nevertheless, this intuitive framework to model different components of multiple omics data illustrates one attempt to meet the promise of network medicine research [88]. The field seeks to integrate biomedical big data and uncover clinically relevant biology by identifying the pathways inside of the disease network rather than isolated components of it. Therefore, in our study, the combination of seeded genes along with the miRNAs with a network of co-expression patterns provided us with a new biological network and insight into relevant mechanisms in CAVD.

## 5. Conclusions

In conclusion, the analysis presented in this study demonstrates that the integration of publicly available multi-omic datasets can be a powerful tool to reveal novel pathways in CAVD. The combination of seeded genes, along with the miRNAs with a network of co-expression patterns, revealed a previously undescribed network in CAVD that is linked to the formation of deposits with amyloid-like properties. Moreover, the coagulation/complement and platelet activation/degranulation pathways seem to play a central role in the pathophysiology of CAVD, and focused research into the related mechanisms may prompt the identification of relevant therapies. The identification of these novel pathways in our study provides a rationale for future mechanistic studies to better understand the pathophysiology of CAVD, as such paving the way for the development of therapeutic strategies.

## Figures and Tables

**Table 1:** Baseline characteristics.

	Total		miRNA		mRNA		Protein	
Studies, *n*	20		5		5		10	
	Control	CAVD	Control	CAVD	Control	CAVD	Control	CAVD
Samples, *n*	270	258	56	52	29	34	185	172
Samples used for omics, *n*	197	172	56	50	29	32	112	90
Age ^1^, mean ± SD	59.0 ± 10.8	68.3 ± 8.2	51.9 ± 11.7	67.8 ± 11.8	50.9 ± 6.1	63.3 ± 5.1	65.7 ± 7.5	70.8 ± 7.2
*p*-value	*p* = 0.008		*p* = 0.105		*p* = 0.021		*p* = 0.166	
Male ^1^, % ± SD	69.8 ± 25.6	67.3 ± 26.1	81.3 ± 23.9	80.8 ± 22.3	91.7 ± 16.7	85.0 ± 30.0	53.2 ± 19.4	53.4 ±19.3
Source of calcified AV, studies/overall studies								
AV replacement surgery		14/20		4/5		5/5		5/10
Source of control AV, studies/overall studies								
Aortic regurgitation	3/20		1/5		2/5			
Autopsy	5/20		1/5		1/5		3/10	
Transplantation	4/20		2/5		2/5			
Non-calcified tissue part	2/20						2/10	
Source of plasma, studies/overall studies								
Patients with AS		6/20		1/5				5/10
Subjects without CVD	5/20		1/5				4/10	
Aortic regurgitation, non-AS	1/20						1/10	

Mean ± standard deviation (SD), CAVD vs. control by unpaired *t*-test. CAVD: calcific aortic valve disease, AV: aortic valve, CVD: cardiovascular disease, AS: aortic stenosis. ^1^ Age and gender not reported by two studies [15,18].

## Supplementary Materials

The following data are available online at https://www.mdpi.com/2073-4409/9/10/2164/s1, Figure S1: Literature search workflow. Figure S2. Pie charts. Figure S3. KEGG pathway diagram highlighting differentially expressed genes/proteins in coagulation and complement cascade pathway from multi-omics datasets in CAVD. Figure S4. Reactome pathway diagram displaying differentially expressed genes/proteins in platelet activation pathway from multi-omics datasets in CAVD. Figure S5. The multi- omics layered network from differentially expressed molecules in CAVD. Figure S6. Subcellular localization of the molecules from the 3D multi-omics layered network. Figure S7. Tissue and plasma-based multi-omics layered network in CAVD. Figure S8. Non-diseased aortic valves from donation did not show expression of transthyretin (TTR) and amyloid precursor protein (APP)/β-amyloid. Table S1. Characteristics of included studies for miRNA. Table S2. Characteristics of included studies for transcriptomics. Table S3. Characteristics of included studies for proteomics. Table S4. Baseline characteristics of study population. Table S5. Differentially expressed molecules. Table S6. miRNA pathway over-representation analysis (DIANA-TarBase). Table S7. mRNA pathway over-representation analysis (ConsensusPathDB). Table S8. Proteome pathway over-representation analysis (ConsensusPathDB). Table S9. Tissue proteome pathway over-representation analysis (ConsensusPathDB). Table S10. Plasma proteome pathway over-representation analysis (ConsensusPathDB). Table S11. Combined proteome and transcriptome differentially expressed genes in CAVD pathway over-representation analysis (ConsensusPathDB). Table S12. Pathway analysis for the 9 common molecules between the proteome and transcriptome datasets (ConsensusPathDB). Table S13. KEGG pathway analysis for multi-omics layered network (miRNA, mRNA, protein; OmicsNet). Table S14. Reactome pathway analysis for multi-omics layered network (miRNA, mRNA, protein; OmicsNet). Table S15. Description of the protein–protein interaction (PPI) connector genes within the layered network from the 9 common genes between transcriptome and proteome and microRNAs. The table summarizes the function of the genes in CAVD. In case no relevant information was found in CAVD, the function of the gene is reported for other diseases. Table S16. KEGG pathway analysis for multi-omics layered network containing the nine common genes between the transcriptome and proteome together with microRNAs in the PPI (OmicsNet). Table S17. Reactome pathway enrichment analysis for multi-omics layered network containing the nine common genes between the transcriptome and proteome together with microRNAs in the PPI (OmicsNet). Table S18. KEGG pathway analysis for multi-omics layered network containing the plasma genes of proteome together with microRNAs in the PPI (OmicsNet). Table S19. Reactome pathway analysis for multi-omics layered network containing the plasma genes of proteome together with microRNAs in the PPI (OmicsNet). Table S20. KEGG pathway analysis for multi-omics layered network containing the tissue genes of proteome together with microRNAs in the PPI (OmicsNet). Table S21. Reactome pathway analysis for multi-omics layered network containing the tissue genes of proteome together with microRNAs in the PPI (OmicsNet).
